# Non-operative Management of Sarcina ventriculi-Associated Severe Emphysematous Gastritis: A Case Report

**DOI:** 10.7759/cureus.31543

**Published:** 2022-11-15

**Authors:** Tyler Birkholz, Grace J Kim, Hannah Niehaus, Kristen Conrad-Schnetz

**Affiliations:** 1 Department of Internal Medicine, Ohio University Heritage College of Osteopathic Medicine, Warrensville Heights, USA; 2 Department of Surgery, South Pointe Hospital Cleveland Clinic Foundation, Warrensville Heights, USA

**Keywords:** non-operative management, gastric perforation, pneumatosis, emphysematous gastritis, gastric ischemia, clostridium ventriculi, sarcina ventriculi

## Abstract

We report a case of a 64-year-old Jehovah's Witness male, who was post-operative day five of laparoscopic cholecystectomy. He presented with anemia, severe ischemic gastritis, and pneumatosis seen on CT with intravenous contrast. A subsequent upper endoscopy revealed patchy gastric ulceration with bleeding but no overt evidence of perforation. Biopsies were taken, and immunohistological staining identified *Sarcina ventriculi*. The patient was treated non-operatively with fluconazole and piperacillin-tazobactam for the infection and with sucralfate tablets and pantoprazole injections for ulcer treatment. After five days, a repeat CT scan revealed a resolved pneumatosis*. S. ventriculi* is a rare bacterium that is increasingly being reported as a cause of emphysematous gastritis with potentially fatal perforation. Surgical intervention should be reserved for unstable patients with perforations and significant, overt bleeding. In this case, non-operative treatment with antibiotics and proton pump inhibitor (PPI) medications were preferred in the setting of anemia in a Jehovah’s Witness patient without perforation. The patient showed clinical and radiologic improvement. Further understanding of the role of surgical intervention versus non-operative management is needed for this rare and potentially life-threatening organism.

## Introduction

*Sarcina ventriculi*, also known as *Clostridium ventriculi*, is a non-motile, anaerobic, Gram-positive coccus [1]^ ^with distinct morphological features, including a cuboid shape, tetrad arrangement, and basophilic staining [2]. Due to its survivability in low pH environments [3], this bacterium has been implicated in fatal epizootic, neurologic, and gastroenteric syndromes in chimpanzees [4] and acute gastric dilatation (AGD) in canines and equines [5].

There are around 65 reported cases of *Sarcina ventriculi *in humans [1] that have been associated with emphysematous gastritis [6-9], delayed gastric emptying [10], and fatal gastric perforation [11]. The exact pathogenesis is still unclear. However, concomitant conditions such as gastric outlet obstruction or delayed gastric emptying may promote the overgrowth of this bacteria, and the presence of concurrent gastric ulcers may lead to emphysematous gastritis or perforation [12].^ ^We are reporting a case of gastric *Sarcina ventriculi *of a Jehovah’s Witness patient with chronic anemia and a recent laparoscopic cholecystectomy who presented with concerns for ischemic and emphysematous gastritis.

## Case presentation

A 64-year-old male, post-operative day five of laparoscopic cholecystectomy for acute on chronic cholecystitis, presented to the emergency department with epigastric pain, frequent bowel movements, nausea, and non-bilious emesis. The patient had a medical history significant for hypertension, poorly controlled insulin-dependent diabetes mellitus type 2, and chronic anemia with an ASA 3 classification in the setting of being a Jehovah’s Witness who refused acceptance of any blood products.

During the initial evaluation, he admitted to having epigastric pain for at least one year prior to this admission. He denied fevers, chills, hematemesis, hematochezia, or chest pain. Physical exam findings on presentation included a heart rate of 118 and a blood pressure of 98/54 that responded to fluids, diffuse abdominal tenderness with voluntary guarding, and healthy wound sites from the laparoscopic surgery. There were no additional abnormal findings. His initial laboratory work-up is listed in Table 1.

**Table 1:** Laboratory results on admission

Laboratory Test	Value
White Blood Cell	14.34k/uL
Hemoglobin	6.7g/dL
Hematocrit	24.1%
Platelet Count	400k/uL
International Normalized Ratio (INR)	1.1
HbA1c	9.3

**Figure 1:**
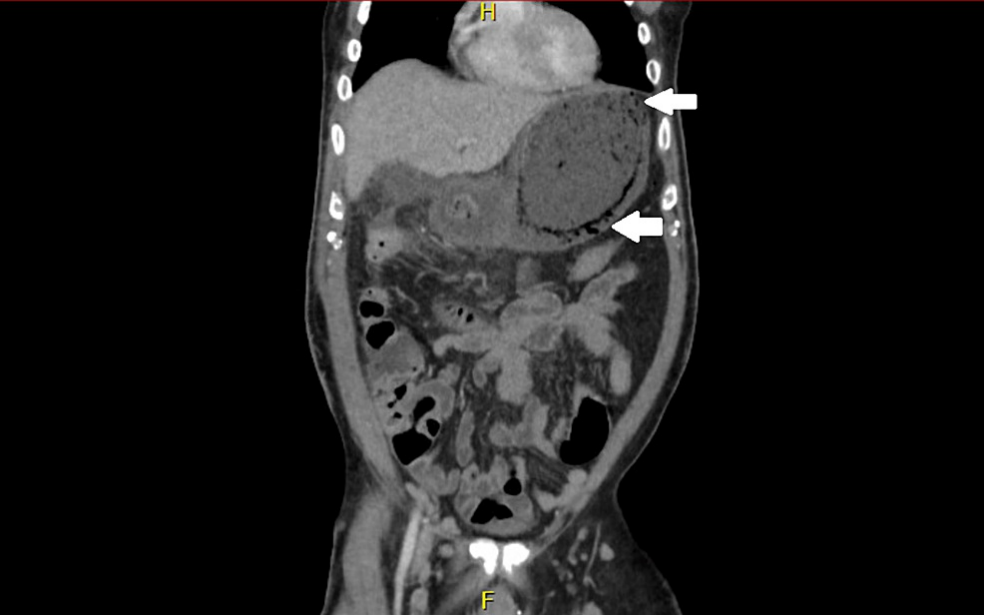
CT scan image of abdomen and pelvis demonstrating severe gastritis with pneumatosis and delayed emptying of the stomach

On hospital day one, CT of the abdomen and pelvis with IV contrast revealed gastric pneumatosis throughout the stomach suggesting either ischemia or necrosis (Figure 1). The general surgery team was consulted and recommended admission for further evaluation and management.

**Figure 2:**
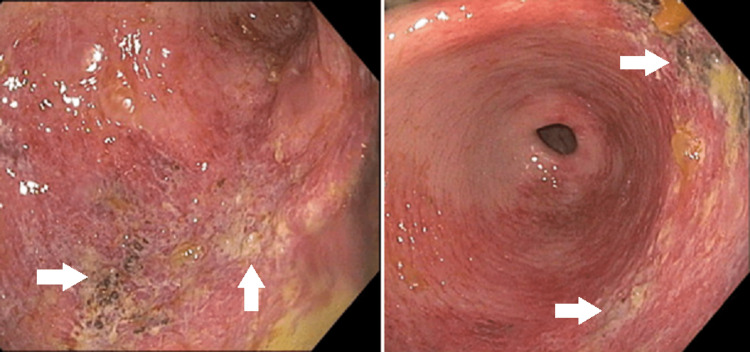
Upper endoscopy demonstrating patchy ulceration of the stomach

**Figure 3:**
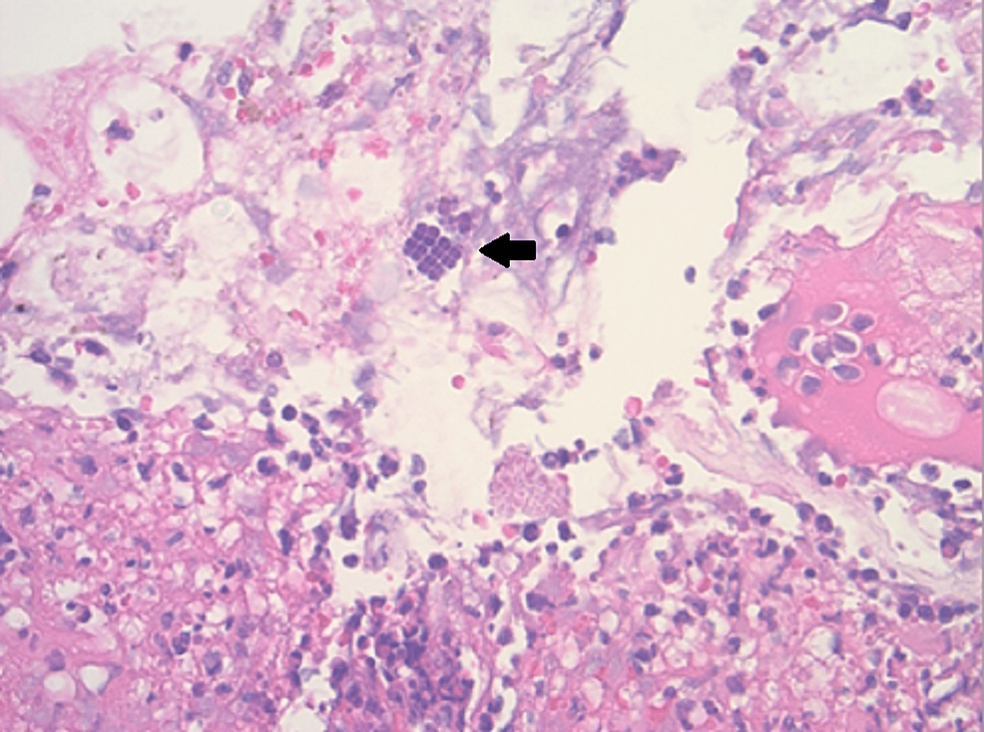
Pathology results from esophagogastroduodenoscopy (EGD) biopsy showing distinct morphological features of Sarcina ventriculi such as a cuboid shape, tetrad arrangement, and basophilic staining

Due to the patient’s religious beliefs, anemia, and lack of free intraperitoneal gas, the surgical team recommended initial non-operative management. An esophagogastroduodenoscopy (EGD) was performed on the day of admission, revealing patchy, friable ulceration without perforation in the antrum of the stomach, lesser curvature, and angular incisura (Figure 2). Tissue biopsies were taken and later revealed no evidence of intestinal metaplasia, dysplasia, or immunohistochemical staining for *Helicobacter pylori*. However, *Sarcina ventriculi* was identified within the acute inflammatory exudate on routine hematoxylin and eosin staining (Figure 3).

**Figure 4:**
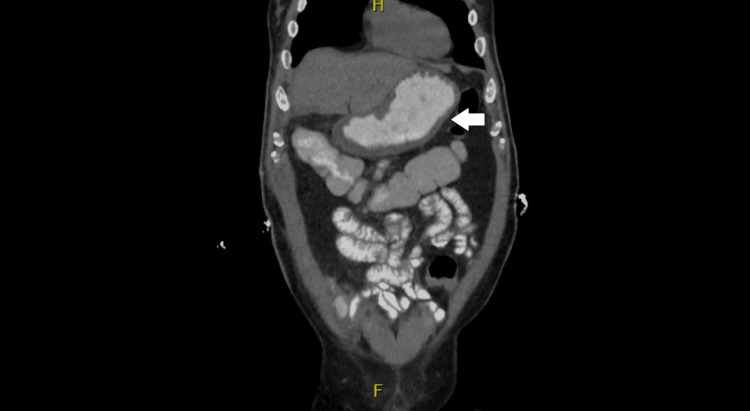
CT abdomen and pelvis showing resolution of gastric pneumatosis after six days of antibiotics and supportive care

For his chronic anemia and refusal of blood product transfusion, erythropoietin 20,000 units subcutaneous injections and IV ferric gluconate 125mg were given. When he tolerated oral medications, his IV ferric gluconate was switched to oral ferric gluconate 325mg. The patient’s hemoglobin remained stable, albeit low, between 5.8g/dL to 7.6g/dL. The patient was treated empirically with IV piperacillin-tazobactam 3.375g, IV clindamycin 600mg, IV vancomycin 1.25g, and IV fluconazole 400mg for his leukocytosis. His antibiotic regimen was de-escalated on hospital day two per the Infectious Disease physician to fluconazole and piperacillin-tazobactam for the duration of his hospital course to cover broadly for upper gastrointestinal (GI) microbes. He also received daily sucralfate 1g tablets and pantoprazole 40mg injections for ulcer treatment. When he tolerated oral intake, the pantoprazole was changed to 40mg tablets twice daily. Serial abdominal exams showed improvement in abdominal pain. His white blood cell count responded to the antibiotics and decreased from a peak of 15.09k/uL to 7.63k/uL. Given the improvement in his clinical picture, he was started on a clear liquid diet on hospital day four, which he did not tolerate. This coupled with a concern for his overall poor nutritional status prompted the placement of a peripherally inserted central catheter line and initiation of total parenteral nutrition (TPN). On hospital day five, a repeat CT scan of the abdomen and pelvis with oral contrast showed interval improvement of gastric wall thickening and mural edema, with interval resolution of pneumatosis (Figure 4).

With antimicrobials and supportive care, the patient began to clinically improve with full resolution of his abdominal pain, nausea, and vomiting. After three days of TPN, he was tolerating a low-fiber diet with adequate oral intake for two days, and his TPN was weaned off. He was discharged on hospital day eleven. Hemoglobin remained stable at 7.6g/dL on discharge. He went home with oral sucralfate and pantoprazole in addition to oral amoxicillin-clavulanic acid 1000mg twice daily for seven days per the Infectious Disease physician's recommendations. He chose to not follow up as an outpatient but reported continued resolution of symptoms over the phone after discharge at both one month and six months.

## Discussion

The pathogenic role of *Sarcina ventriculi* in humans is not entirely understood despite an increasing number of reported cases. It has been isolated in blood [13,14], as well as esophageal, pyloric and duodenal brushings [15-16], fine-needle aspirations of the stomach [17], and tissue biopsies of the stomach [18]. It has also been implicated in esophageal stricturing [19] along with emphysematous gastritis and fatal perforations. However, there have also been cases of asymptomatic patients with *Sarcina *found on biopsies [17,20]. How *Sarcina *over-proliferates in the body or why it causes fatal complications in some individuals and not others need to be further elucidated. Some of the cases cited in this report have also indicated delayed gastric emptying either from diabetes or gastric surgery, immunocompromised state, and concomitant *Candida* infections in their patients with *Sarcina*, which may play a role in its growth [1,10,21]. In our patient, delayed gastric emptying likely played a role in his complications judging by the gastric contents still seen during the EGD.

A systematic review of the literature for *Sarcina *infections showed that out of 66 reported cases, about 53% received antimicrobial therapy and 20% of the cases received proton pump inhibitors (PPIs) with 88% eradication on follow-up EGD. Surgical treatment was performed in 23% of cases for gastric perforation, necrosis, stenosis, and massive pneumoperitoneum [1]. Surgical interventions included partial gastrectomy, fundoplication and hiatal hernia repair, perforation primary repair, jejunostomy, ileocecal resection with ileostomy, and fistula excision [1]. What is also unclear in our patient is the role of his recent surgery and the etiology of gangrenous cholecystitis in this subacute presentation post-operatively. Tokyo Guidelines 2018 recommend that patients with grade II and III acute cholecystitis should have bile cultures obtained to help guide anti-microbial administration [22]. Our patient had grade II cholecystitis, and we did not obtain cultures for our patient since continuing antibiotics post-operatively was not necessary. If the patient had grade III acute cholecystitis or acute cholangitis, the guidelines recommend extending antimicrobial therapy to 4-7 days after source control. In this case, an argument can be made to obtain bile cultures at the time of surgery to direct antimicrobial therapy and possibly prevent further infection.

## Conclusions

Further research is needed to understand the indications and contraindications for surgical and non-surgical intervention of *Sarcina *infections. In clinically stable patients, non-operative management with serial abdominal exams, nutritional support, optimization of intestinal motility, and pharmacologic administration may be sufficient. Further research into an ideal antimicrobial regimen and timing of bowel rest is needed, as different case reviews offer varying options. Our patient showed radiographic and clinical improvement with non-operative management, which coincides with the majority of cases reported in the literature.
